# Development of a Zika Virus Infection Model in Cynomolgus Macaques

**DOI:** 10.3389/fmicb.2016.02028

**Published:** 2016-12-19

**Authors:** Fusataka Koide, Scott Goebel, Beth Snyder, Kevin B. Walters, Alison Gast, Kimberly Hagelin, Raj Kalkeri, Jonathan Rayner

**Affiliations:** ^1^Department of Infectious Disease Research, Drug Development, Southern Research Institute, FrederickMD, USA; ^2^Department of Infectious Disease Research, Drug Development, Southern Research, BirminghamAL, USA

**Keywords:** Zika virus, cynomolgus macaque, non-human primate, flavivirus, arbovirus

## Abstract

Limited availability of Indian rhesus macaques (IRM) is a bottleneck to study Zika virus (ZIKV) pathogenesis and evaluation of appropriate control measures in non-human primates. To address these issues, we report here the Mauritian cynomolgus macaque (MCM) model for ZIKV infection. In brief, six MCMs (seronegative for Dengue and ZIKV) were subdivided into three cohorts with a male and female each and challenged with different doses of Asian [PRVABC59 (Puerto Rico) or FSS13025 (Cambodia)] or African (IBH30656) lineage ZIKV isolates. Clinical signs were monitored; and biological fluids (serum, saliva, and urine) and tissues (testes and brain) were assessed for viral load by quantitative reverse transcription polymerase chain reaction and neutralizing antibodies (Nab) by 50% Plaque Reduction Neutralization Test (PRNT_50_) at various times post-infection (p.i). PRVABC59 induced viremia detectable up to day 10, with peak viral load at 2–3 days p.i. An intermittent viremia spike was observed on day 30 with titers reaching 2.5 × 10^3^ genomes/mL. Moderate viral load was observed in testes, urine and saliva. In contrast, FSS13025 induced viremia lasting only up to 6 days and detectable viral loads in testes but not in urine and saliva. Recurrent viremia was detected but at lower titers compare to PRVABC59. Challenge with either PRVABC59 or FSS13025 resulted in 100% seroconversion; with mean PRNT_50_ titers ranging from 597 to 5179. IBH30656 failed to establish infection in MCM suggesting that MCM are susceptible to infection with ZIKV isolates of the Asian lineage but not from Africa. Due to the similarity of biphasic viremia and Nab responses between MCM and IRM models, MCM could be a suitable alternative for evaluation of ZIKV vaccine and therapeutic candidates.

## Introduction

Zika virus (ZIKV) is a flavivirus transmitted primarily through mosquitos, first reported in 1947 (reviewed in Plourde and Bloch, 2016). Though it was initially restricted to Africa and Asia, in the last couple of years increasing cases have been observed in as many as 70 countries worldwide. Though apparent clinical symptoms are reported only in 20% of the infected patients, the disease associated complications such as microcephaly in newborn children and neurological manifestations (Guillain–Barre syndrome) make ZIKV a major health concern. Considering the rapid spread and the associated disease complications, the World Health Organization (WHO) has recently declared ZIKV as a global health emergency. This health scare is compounded by the lack of effective prophylactic and therapeutic measures.

Currently, immune compromised mouse (Brault et al., 2016; Cugola et al., 2016; Dowall et al., 2016; Lazear et al., 2016; Zmurko et al., 2016) and rhesus macaque models (Abbink et al., 2016; Dudley et al., 2016) have been used for studies on the natural history and pathogenesis of ZIKV infection. Type-I interferon receptor deficient AG129 mice but not the parent 129Sv/Ev strain of mice were found to be susceptible to a lethal ZIKV infection (Dowall et al., 2016). Lazear et al. (2016) reported the development of neurological disease in IFNar1 (-/-) mice and IRF3, 5, and 7 triple knockout mice (Lazear et al., 2016). The AG129 model was also helpful in evaluating the antiviral activity of viral polymerase inhibitor 7-deaza-2′-C-methyladenosine (7DMA) (Zmurko et al., 2016). Using the Swiss Jim Lambert (SJL) mouse model, Cugola et al. (2016) were able to demonstrate fetal infection and microcephaly with a Brazilian strain of Zika virus. Though mouse models are easily accessible, non-human primates (NHPs) are an attractive model for ZIKV research and drug discovery due to their close similarity with humans. NHP models could provide invaluable information regarding mechanism of action, efficacy and safety of both drug and vaccine candidates and allow optimization of the product, dose and route as observed previously for HIV vaccines (Spearman, 2006). Rhesus macaques were shown to be susceptible to an Asian lineage of ZIKV (Dudley et al., 2016), with pregnant animals being viremic for longer period compared to non-pregnant animals. Evaluation of three vaccine candidates in rhesus monkeys successfully protected them against ZIKV challenge (Abbink et al., 2016).

The use of a number of non-rhesus macaque species, especially cynomolgus macaques, as a model for human infectious diseases has increased in recent years (Antony and MacDonald, 2015). This is mostly due to the reduced availability of Rhesus monkeys consequent to the ban on their export from India. Compared to Rhesus macaques, Cynomolgus macaques offer the advantages of smaller size and weight (Andrade et al., 2004), resulting in reduced amounts of drugs needed for studies administered on body weight basis. Smaller animal size also provides the additional benefit of easier animal husbandry practices (such as handling, space requirements, etc.), translating into significant cost-benefit. Considering these factors, we conducted a limited study (*N* = 2/group) to evaluate the suitability of cynomolgus monkeys as a potential alternative NHP model for ZIKV infection. Using a systematic approach of infection with ZIKV strains of different geographical origin, we demonstrate that cynomolgus monkeys can be successfully infected with ZIKV of Asian-lineage including isolates recently emerging in the current pandemic of the Americas, but not strains of African lineage.

## Materials and Methods

### Care and Use of Animals

This study was designed to use the fewest number of animals possible, consistent with the objective of the study, the scientific needs, contemporary scientific standards, and in consideration of applicable regulatory requirements. This study design was reviewed by the IACUC at Southern Research Institute and was approved on 04/21/2016; it was assigned IACUC tracking number 16-03-014F. Animals were socially housed during the quarantine phase and single housed following the Day 0 challenge. Animals were housed in stainless steel cages that meet requirements as set forth in the Animal Welfare Act (Public Law 99-198) and the *Guide for the Care and Use of Laboratory Animals* (8th Edition, Institute of Animal Resources, Commission on Life Sciences, National Research Council; National Academy Press; Washington D.C.; 2011). Animals were housed in an environmentally monitored and ventilated room. Fluorescent lighting provided illumination approximately 12 h per day. The objective of this pilot proof of concept study was to evaluate the susceptibility of cynomolgus macaques to ZIKV of different geographic origins and did not involve statistical comparison between groups of animals. We’ve selected two monkeys per challenge strain and this is deemed sufficient to provide enough data to monitor immunological and virological endpoints against each strain. The use of two animals per strain is the minimum number sufficient to achieve the research goals. Based on the results obtained from this pilot study, statistically relevant sample size will be determined for future GLP studies.

### Orchiectomy Surgery

The animals were initially given either atropine (0.02–0.04 mg/kg IM) to control respiratory secretions, then sedated with ketamine (10–50 mg/kg IM). Ketamine was followed by xylazine (0.30 mg/kg IM) for induction. The animals were then intubated, placed on a portable isoflurane machine, and isoflurane (0.5–5.0%) was used to bring the animals to the desired plane of anesthesia for the procedure. Anesthesia was maintained using approximately 1–3% isoflurane throughout the procedure. Before the initial incision, ketoprofen (2.2 mg/kg IM, SID) and buprenorphine (0.01–0.03 mg/kg IM, BID) were administered. A lidocaine/bupivacaine local block (at no more than 1.0 mg/kg of each agent) was given at the incision area before surgery began. Orchiectomy was performed to one testes per day. After surgery, animals were removed from the isoflurane machine and placed on towels/blankets with a warming system (Bair-Hugger mat) and monitored until they recovered their swallowing reflex. At this time, the endotracheal tube was removed and the animal was returned to its home cage. Animals were observed continually by trained technicians until they were able to sit upright, at which time they were considered to have recovered from the anesthesia. Ketoprofen (2.2 mg/kg IM, SID) and buprenorphine (0.01–0.03 mg/kg IM, BID) were given as post-operative analgesia for at least 2 days following the day of surgery.

### Sequence Analysis

Comparative ZIKV genomic sequence analysis and alignments were performed using the NCBI BLAST Suite^1^. The “Megablastn, Blastn” programs were used for the genomic comparisons and “Primer-blast” for sequence specific primer homology analysis.

### Viruses and Cell Culture

Vero cells (ATCC, CCL81) were grown in Dulbecco’s Minimal Essential Medium (DMEM, Lonza), supplemented with 10% fetal bovine serum (FBS), NEAA and L-Glutamine according to standard culture conditions. ZIKV strains were obtained from the following sources: IBH30656 (BEI Resources), PRVABC59 (CDC, Division of Vector-borne Infectious Diseases) and FSS13025 (UTMB Arbovirus Reference Collection). In addition, the following ZIKV isolates were used as reference strains for comparative sequence analysis: ZKV2015 (Genbank Accession #KU497555.1) (Calvet et al., 2016), Brazilian isolate Natal RGN, Bahia, Brazil (Seq. Accession# KU527068.1) (Calvet et al., 2016; Mlakar et al., 2016). Viral stocks of each strain were amplified in Vero cells and quantitated according to the standard plaque assay methodology.

### Primers and Probes

Polymerase chain reaction (PCR) primers were designed to the NS1 region of the ZIKV genome, proximal to those described (Lanciotti et al., 2008) with modified target sequences enabling the detection of isolates PRVABC59 Puerto Rico (Asian lineage) and FSS13025 Cambodia (Asian lineage), IBH30656 Nigeria (African lineage). Primer and probe sequences are provided in Goebel et al. (2016). Primer and probe sequences were characterized for compatible melting temperatures (Tm), self-dimer and hairpin potential using the Integrated DNA Technologies (IDT, Coralville, IA) “Primer Analysis software” tool. All the primers and probes were synthesized at IDT Technologies. All primers were subjected to sequence analysis including specific ZIKV strain homology and hybridization potential across different clades. The probe has a 5′ 6-FAM reporter and an internal (9th position) ZEN quencher and a 3′ IBFQ Iowa black quencher. Primers and protocols used for the generation of the RNA template used for the standard curve for absolute quantitation were as described in Goebel et al. (2016).

### Quantitative RT-PCR

Viral RNA was extracted from biological fluid samples (serum, urine, and saliva) and tissue samples, including testes and brain. Blood samples for serum were collected from anesthetized animals on 1–4, 6, 8, 10, 14, 30, and 60 days post-infection (p.i). Urine and oral swab saliva samples were collected on 1–4, 6, 8, 10, 14 days p.i. Urine samples were collected from anesthetized animals by catheterization or cystocentesis. Urine was not successfully collected at all time points, especially from females (indicated in **Table 1**). Saliva samples were obtained by swabbing the oral cavity with a cotton swab, followed by emersion of the swab into 2 mL PBS without Calcium and Magnesium (Lonza, 17-512F). Briefly, using the QIAmp Viral RNA Mini kit (Qiagen, 52906) total RNA was extracted and purified from a total biological sample volume of 140 μl (as per manufacturer) and eluted into 60 μL of nuclease free water (Ambion, AM9939). Total RNA was extracted from tissues samples as per manufacturer using the RNeasy Lipid Tissue mini kit (Qiagen, 74804). Briefly, several (100–150 mg) sections were dissected from each tissue sample. Tissue sections were subjected to mechanical tissue homogenization in 1 mL of QIAzol Lysis Reagent (Qiagen, 1023537) using a handheld tissue homogenizer (Omni International) fitted with a rotor stator. Purified RNA was eluted in 50 μL nuclease free water.

**Table 1:** Virus shedding in urine and saliva.

	Urine						Oral Swabs					
	PRVABC59		FSS13025		IBH30656		PRVABC59		FSS13025		IBH30656	
Day	#5262M	#5258F	#5260M	#5259F	#5261M	#5257F	#5262M	#5258F	#5260M	#5259	#5261M	#5257F
1	0	0	0	N/C	0	N/C	0	0	0	1.6	N/T	N/T
2	265	0	N/C	N/C	43	N/C	105	0	0	0	N/T	N/T
3	0	0	19	N/C	N/C	N/C	198	0	52	0	N/T	N/T
4	0	N/C	N/C	N/C	0	N/C	169	146	0	1	N/T	N/T
6	0	29	0	N/C	0	N/C	0	0	6.6	2	N/T	N/T
8	0	0	N/C	N/C	0	N/C	138	142	0	81	N/T	N/T
10	0	87	0	N/C	200	N/C	244	0	12	0	N/T	N/T
14	0	114	N/C	N/C	0	N/C	109	359	0	0	N/T	N/T

*N/C, not collected; N/T, not tested.*

Five μl of purified RNA from each test article was used in a 20 μl qRT-PCR reaction consisting of Fast Virus 4x Master Mix (Applied Biosystems, 4444436) containing 500 nM forward and reverse primers and 200 nM probe. Cycling parameters included an initial reverse transcription (RT) for 5 min at 53°C, followed by 1 min at 95°C and 45 cycles of two step cycling at 95°C for 5 s and 60°C for 50 s. A standard curve using positive control RNA template was established over a dynamic range of 6-logs (10^6^–10^1^) with each dilution in triplicate for absolute quantitation of test samples. The Ct values obtained for each of the 6 log dilutions indicate each strain specific primer probe set to be consistently within 1 Ct of each other. Furthermore, each of the strain specific primer and probe sets for the qRT-PCR reaction were found to have a very similar lower limit of quantitation (LLOQ) with values of no more than 10 copies/reaction or 500 copies/mL (data not shown).

### Statistics

The mean viral genome copies found in 4 and 8 day p.i. teste samples were compared by one-way analysis of variance (ANOVA) followed by Bonferroni’s multiple comparison test. Differences in means were considered statistically significant for a *p*-value < 0.05.

### Plaque Reduction Neutralization Test (PRNT_50_)

Briefly, Vero cells seeded at a concentration of approximately 3 × 10^5^ cells/mL in 24-well plates were incubated for approximately 24 h. Day of assay, input virus (PRVABC59) and serially diluted serum samples were mixed and incubated for 1 h at 37 ± 1°C in the dilution plate. Supernatant from cell-seeded 24-well plates was decanted, then 100 μl of virus/serum mixture was transferred from the dilution plate to the cells. After 1 h adsorption, agarose-containing overlay media was added and plates were incubated at 37 ± 1°C, 5% CO_2_ for 3 days. The cells were fixed and stained using crystal violet solution and plaques were counted visually. The neutralizing antibody titer was expressed as the highest test serum dilution for which the virus infectivity is reduced by 50%.

## Results

### Cohorts

A total of six cynomolgus macaques, seronegative for Dengue and ZIKV by ELSIA were subdivided in three cohorts, each containing a male and female. Animals were inoculated subcutaneously with ZIKV from either the Asian lineage (Puerto Rico isolate; PRVABC59, or Cambodian isolate; FSS13025) or an African lineage (Nigerian isolate; IBH30656). Male and female animals were inoculated on Day 0 with 5.0 × 10^5^ PFU and 1.0 × 10^4^ PFU, respectively. Previous studies have shown viremia in cynomologus macaques following infection with similar concentrations of Dengue virus. Additionally, natural levels of West Nile Virus (WNV) transmission through a mosquito “bite” reportedly range between 1.0 × 10^4^ and 1.0 × 10^6^ PFU (Styer et al., 2007; Cox et al., 2012). Biological fluids (serum, salvia, and urine) were collected at various times between 1 and 60 days p.i. Selected tissue samples were also collected including testes on days 4 and 8 and brain tissue upon animal sacrifice at study termination on day 60. Samples were assessed for viral load and shedding using reverse transcription followed by quantitative PCR (qRT-PCR) and immunological responses were assessed using the 50% Plaque Reduction Neutralization Test (PRNT_50_).

### Clinical Observations

One animal challenged with IBH30656 developed a slight erythema around the injection site (Draize dermal score of 1) on study days 8 and 10. The erythema had resolved by day 14. No clinical symptoms were observed in any of the other animals during the course of the study.

### Serum Viral Load Analysis

**FIGURE 1:**
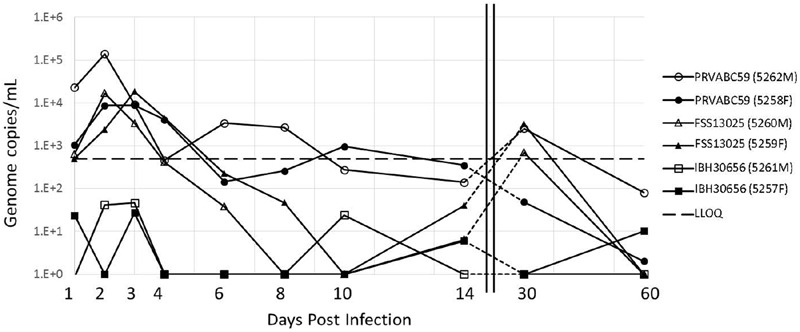
**Viral load in the serum of ZIKV infected cynomolgus macaques.** Animals were grouped into three cohorts, each containing a male (opened symbols) and female (closed symbols). Each cohort was challenged with ZIKV isolates at the dose indicated with either PRVABC59 (●), FSS13025 (▲), or IBH30656 (■). The serum viral load was determined using qRT-PCR at various days p.i. Notably, the serum viral load peaks around day 2–3 p.i. The LLOQ was previously determined to be 500 copies/mL and is indicated by the black dashed line.

Viral load was determined by qRT-PCR in serum recovered from blood collected on days 1–4, 6, 8, 10, 14, 30, and 60. Both cohorts challenged with Asian lineage strains, PRVABC59 or FSS13025, resulted in substantial viral load that peaked between 2 and 3 days p.i. Maximal serum load for the PRVABC59 challenge (high/low dose) was (1.4 × 10^5^/8.9 × 10^3^) genome copies/mL and for FSS13025 (1.7 × 10^4^/1.8 × 10^4^) copies/mL (**Figure 1**). However, the cohort infected with the African lineage strain IBH30656 failed to produce a “productive” infection as viral load peaked 3 day p.i. at less than 100 copies/mL, well below our defined LLOQ detection.

### Viral Clearance

Shortly after peaking at 2 or 3 days p.i., serum viremia diminished over the course of about 10 days depending on the virus. By 14 days p.i. all viral loads were below the LLOQ of the qRT-PCR assay at 500 copies/mL. Interestingly, we observed a consistent viral load rebound of about 2 log by 30 day p.i. in three of the four animals infected with either of the two Asian lineage strains. This viral load rebound from serum samples is consistent with that previously reported from a ZIKV challenge in rhesus macaques (Dudley et al., 2016). In cynomolgus macaques the rebound seems to persist longer, at least through day 30 p.i. which was the only intermediate viral load test point between day 14 and the study termination at day 60. Viremia associated with the later viral load rebound diminished to less than 100 copies/mL by 60 days p.i. (**Figure 1**).

### Viral Shedding in Body Fluids

In contrast to the robust amounts of virus found in the serum of animals challenged with the Asian lineage viruses (PRVABC59 and FSS13025), saliva and urine samples produced moderate levels of viral load (<300 copies/mL). Lower viral loads in saliva may be partly due to the dilution of swabs in PBS prior to analysis. As expected, considering the serum viral load, the animals challenged with the African strain IBH30656 resulted in extremely low, sporadic amounts of detectable viral RNA levels (**Table 1**).

### Tissue Analysis

**FIGURE 2:**
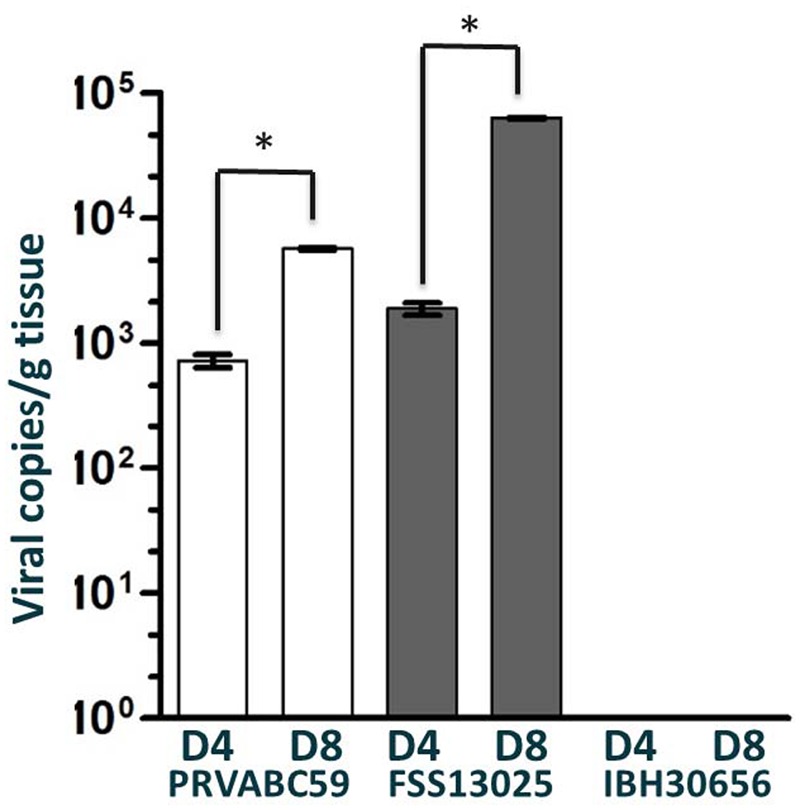
**Viral load in the testes of ZIKV infected cynomolgus macaques.** Viral load in the testes of cynomolgus macaques infected with PRVABC59, FSS13025, or IBH30656 were detected by qRT-PCR on days 4 and 8 p.i. Multiple sections of each testicle were tested independently and reported here are the sections with the highest viral load. ^∗^*p*-value < 0.05.

One testicle was harvested at 4 and 8 days p.i. from the male of each cohort. The highest viral loads were consistently detected from testes samples collected on day 8 post-inoculation. Interestingly, the FSS13025 challenged male had the highest testes viral load (**Figure 2**). Again consistent with the serum analysis, no viral load was detected in the testes of animal challenged with the African strain IBH30656. Upon study termination (60 days p.i.) total brain samples were harvested from all animals. Multiple tissue sections from each brain were tested for viral load by qRT-PCR and all were negative for any residual viral genome copies.

### Neutralizing Antibody Response

Neutralizing antibodies (Nabs) following Zika challenge are illustrated in **Table 2**. Sera collected on day 0 prior to challenge were all negative (PRNT_50_ ≤ 10). The IBH30656 challenged monkeys (5261 and 5257), that exhibited very low viral load throughout study period, also failed to produce cross reactive Nab to PRVABC59. The monkeys inoculated with the PRVABC59 and FSS13025 achieved high Nab titer to PRVABC59 by day 14. Monkey 5262 that recorded highest viremia also attained highest Nab titer of 7920. The day 14 Nab titers for these monkeys ranged from 1,053 to 7,920. All four monkeys suffered a moderate drop in titer on day 30 but Nab activity was persistent and achieved titers ranging from 797 to 2,380. Notably, monkey 5262 (PRVABC59) and 5259 (FSS13025), that experienced more apparent viremia rebound by 30 days p.i., attained >2-fold higher Nab response than the other monkey in the same cohort during this time. Nab titer was sustainable and all four macaques achieved durable PRNT_50_ titers ranging from 528 ≥ 1,071 at 60 days p.i.

**Table 2:** Development of anti-zika neutralizing antibodies following challenge.

Animal ID	Challenge strain	Dose (PFU)	PRNT_50_ Titer			
			D2	D14	D30	D60
5262 M	PRVABC59	5.0 × 10^5^	<10	7920	2380	1071
5258 F	PRVABC59	1.0 × 10^4^	<10	2438	875	740
5260 M	FSS13025	5.0 × 10^5^	<10	1053	797	528
5259 F	FSS13025	1.0 × 10^4^	<10	3131	2019	666
5261 M	IBH 30656	5.0 × 10^5^	<10	<10	<10	<10
5257 F	IBH 30656	1.0 × 10^4^	<10	<10	<10	<10

*Anti-PRVABC59 neutralizing antibodies were measured in serum collected at 2, 14, 30, and 60 days p.i. by standard PRNT assay. PRNT end-point titers are expressed as the reciprocal of the last serum dilution showing the 50% reduction in plaque counts (PRNT_50_).*

## Discussion

Arthropod-borne viruses such as the flaviviruses continue to pose a significant threat to global health. The origin and global spread of ZIKV during the ongoing pandemic is well-documented (Dick et al., 1952; Duffy et al., 2009; Haddow et al., 2012; Musso et al., 2014; Campos et al., 2015; Zanluca et al., 2015; Solomon et al., 2016). The significance of ZIKV is highlighted in the infection associated complications, such as Guillain–Barre syndrome (Oehler et al., 2014; do Rosario et al., 2016) and the risk of vertical transmission of the virus from mother to the fetus, which can result in devastating lifelong neurological complications including microcephaly (Brasil and Nielsen-Saines, 2016; Martines et al., 2016; Mlakar et al., 2016; Sarno et al., 2016). Considering the global health emergency posed by ZIKV, there is an ongoing concerted effort among the scientific community to develop new diagnostics, vaccines and antivirals to stem the pandemic. Identification of cost effective and robust animal models amenable to measuring viral load, viral shedding and building cellular and humoral immune correlates of vaccine efficacy is critical for the development of new vaccines and therapeutics. Here, we report the results from a limited study demonstrating the utility of cynomolgus macaques as a cost effective alternative NHP animal model for the study of ZIKV infection of Asian lineage.

Failure of the African strain IBH30656 to produce a significant viremia is in contrast to the previous work done in rhesus macaques by the Wisconsin National Primate Research Center^2^, where rhesus macaques developed a robust viremia when challenged with the prototypic African strain, Uganda MR766 (GenBank, LC002520, obtained from CDC, Ft. Collins, CO, USA). In addition to the differences in animal models, potentially significant genetic differences between the two African lineage strains may exist. These differences may encode changes leading to altered codon utilization or post-translation processing which have been proposed to play a role in ZIKV pathogenicity (Haddow et al., 2012; Gupta et al., 2016; van Hemert and Berkhout, 2016). Indeed, these two isolates have differences in passage history and share only 93% identity at the nucleotide level. Heterogeneity between available MR766 sequences makes genetic comparisons with IbH30656 difficult (Haddow et al., 2012). Additional studies are needed to further characterize the host and viral factors involved in divergent pathogenicity between ZIKV strains.

Interestingly, the Puerto Rican isolate PRVABC59 appeared to provide the most robust infection, resulting in rapid systemic serum viral load within the first 24 h p.i. (2.3 × 10^4^ and 1.1 × 10^3^ copies/mL) compared to the Cambodian FSS13025 isolate (6.4 × 10^2^ and 5.0 × 10^2^ copies/mL). The temporary lag in amplification of the FSS13025 isolate may suggest different replication kinetics between these two strains. In the rhesus macaque model, peak plasma viremia occurred between 2 and 6 days p.i. and peak virus titer ranged from 8.2 × 10^4^ to 2.5 × 10^6^ RNA copies/mL after 1.0 × 10^4^ to 1.0 × 10^6^ challenge with Asian strains (Dudley et al., 2016). Altogether, our study shows the peak viral load was moderately lower in the cynomolgus macaque, the duration and overall kinetics of virus replication produced by Asian strains were similar to those observed in the rhesus monkeys.

Recent bioinformatics and phylogenetic analyses, have suggested the possible derivation of the currently circulating (in the Americas) ZIKV pandemic isolates from the Asian lineage (Brasil et al., 2016; Calvet et al., 2016; Lanciotti et al., 2016). Furthermore, the sequences of clinical samples isolated from the American outbreak are highly conserved, and are therefore now recognized as a new clade of the Asian lineage (Enfissi et al., 2016; Wang et al., 2016; Ye et al., 2016). Using genomic sequence alignments, a comparison between historic Asian lineage isolates, currently circulating American clade isolates and original African lineage isolates demonstrates how divergent the American clade has become from the African lineage (**Table 3**). In our study, infection of NHPs with the African lineage virus (IBH30656) did not elicit an immune response that cross-neutralized to PRVABC59. This may be due to antigenicity difference between African and Asian lineage viruses but most likely is due to the failure of the virus to establish a productive infection. Numerous studies have now been published on comparative genomics and epidemiological analysis aimed at identifying critical adaptive mutation(s) or processes such as codon utilization or glycosylation that may contribute to the pathogenicity and/or fitness in transmission of the emergent American clade (Gupta et al., 2016; van Hemert and Berkhout, 2016; Ye et al., 2016).

**Table 3:** Genomic sequence comparison of ZIKV isolates.

*ZIKV Strain: Lineage/clade:*	*MR-766 African*	*IBH30656 African*	*FSS13025 Asian*	*PRVABC59 Asian*	*ZKV2015 American*
*MR-766*	----	93	89	89	89
*IBH30656*	93	----	88	88	88
*FSS13025*	89	88	----	98	98
*PRVABC59*	89	88	98	----	99
*ZKV2015*	89	88	98	99	----

*Pairwise comparison of nucleotide sequence using NCBI (https://blast.ncbi.nlm.nih.gov) Blastn sequence alignment tool. Strains of African and Asian lineage were aligned with the newly emerging American Clade (Ye et al., 2016). ZKV2015 (Accession #KU497555.1) represents the American clade, the most clinically relevant strains circulating in Brazil.*

Broad systemic infection of cynomolgus macaques with two independent Asian lineage ZIKV isolates including one from the current circulating pandemic, observed in our studies, suggest the clinical relevance of this model. The failure to detect systemic infection after challenge with the the Nigerian IBH30656 strain of African lineage, is confounding, as other strains of the African lineage (MR766) have been used in rhesus macaques and found to produce robust infections in that animal model as stated above. Whether this observation is limited to Nigerian IBH30656 or more broadly, to other African isolates in cynomolgus macaques need to be explored further.

Additionally, it would be of great interest to elucidate the nature of the late recurrent viremia, now consistently seen in both the cynomolgus and rhesus models reported previously (Dudley et al., 2016). Although this “rebound virus” is eventually cleared in the NHP animal models discussed, the identification of tissue specific reservoir(s) for the rebound virus could lead to mechanisms posing additional adaptive risks and changes in the progression of the infection in humans, either through enhanced transmission or potentially escalating clinical complications.

In the context of viral reservoirs, it is interesting to note that the viral load of both PRVABC59 and FSS13025 in the testes at 8 days p.i. were higher in comparison to 4 days p.i. Importantly, this is well after the serum viral load had peaked between 2 and 3 days p.i. This observation is consistent with previously reported that male anatomical tissues have provided safe harbor for persistent viral titers, long after symptoms and or systemic viral load have resolved (Osuna et al., 2016).

Interestingly, Dudley et al. (2016) suggests that the viral plasma “blips” seen after systemic clearance maybe the result of viral seeding from these reservoirs. Importantly, understanding tissue specific viral reservoirs and their role in providing for mechanisms of viral adaptive immune escape would be critical to the understanding of non-arthropod vectored transmission in the current pandemic strain, and may provide strategic insights for the development of new vaccines and therapeutics.

In this study, detection of serum and tissue viral load was associated with the development of high functional Nab titers in cynomolgus macaques infected with PRVABC59. These results provide insight into the possibility of cynomolgus macaques as an alternative model to study vaccine immunogenicity and efficacy to decipher correlates of protective immunity. In light of possible Zika-Dengue bi-directional antibody-dependent enhancement (ADE) of disease (Dejnirattisai et al., 2016), availability of surrogate animal models of both viruses to predict safety and clinical benefit of candidate vaccines is essential. Cynomolgus macaques have been established as a model for Dengue virus infection and are currently be used for preclinical safety and efficacy of vaccine candidates. The objective of this study was to establish proof of concept Zika natural history data in a limited number of cynomolgus monkey’s to justify continued model development including an ADE model. Taken together, our preliminary data supports continued development of cynomolgus macaques as a model for ZIKV infection and certainly warrant further investigation with more statistically relevant numbers of animals. A cynomolgus macaque model of ZIKV infection could be a useful tool to understand the ZIKV natural infection, pathology and develop effective control measures.

## Author Contributions

FK is the Principal Investigator and Study Director of this study. He designed the study, and authored the study and IACUC protocols. SG developed the RT-qPCR assay, analyzed the results and contributed to manuscript preparation. BS is a scientist who propagated Zika virus and performed Zika virus plaque assays. KW is a scientist who directed virus propagation and characterization, contributed to the protocols and manuscript preparation. AG is a scientist who performed all RNA extractions and contributed to RT-qPCR. KH conducted NHP handling, virus dosing of NHPs, sample collections and necropsy of animals. RK is a scientist who developed the *in vitro* protocols for this project, advised SG and BS and contributed to manuscript preparation. JR is a Co-PI and supported study design and study protocol development. He also supported proposal writing to secure funding for this work.

## Conflict of Interest Statement

The authors declare that the research was conducted in the absence of any commercial or financial relationships that could be construed as a potential conflict of interest.

## Footnotes

1 http://www.ncbi.nlm.nih.gov/pubmed

2 https://zika.labkey.com/project/OConnor/ZIKV-002/begin.view
